# Markov Chain-Based Sampling for Exploring RNA Secondary Structure under the Nearest Neighbor Thermodynamic Model and Extended Applications

**DOI:** 10.3390/mca25040067

**Published:** 2020-10-10

**Authors:** Anna Kirkpatrick, Kalen Patton, Prasad Tetali, Cassie Mitchell

**Affiliations:** 1 School of Mathematics, Georgia Institute of Technology, Atlanta, GA 30332, USA; 2 School of Computer Science, Georgia Institute of Technology, Atlanta, GA 30332, USA; 3 Department of Biomedical Engineering, Georgia Institute of Technology, Atlanta, GA 30332, USA

**Keywords:** Markov chain Monte Carlo, RNA secondary structure, nearest neighbor thermodynamic Model, Markov chain convergence

## Abstract

Ribonucleic acid (RNA) secondary structures and branching properties are important for determining functional ramifications in biology. While energy minimization of the Nearest Neighbor Thermodynamic Model (NNTM) is commonly used to identify such properties (number of hairpins, maximum ladder distance, etc.), it is difficult to know whether the resultant values fall within expected dispersion thresholds for a given energy function. The goal of this study was to construct a Markov chain capable of examining the dispersion of RNA secondary structures and branching properties obtained from NNTM energy function minimization independent of a specific nucleotide sequence. Plane trees are studied as a model for RNA secondary structure, with energy assigned to each tree based on the NNTM, and a corresponding Gibbs distribution is defined on the trees. Through a bijection between plane trees and 2-Motzkin paths, a Markov chain converging to the Gibbs distribution is constructed, and fast mixing time is established by estimating the spectral gap of the chain. The spectral gap estimate is obtained through a series of decompositions of the chain and also by building on known mixing time results for other chains on Dyck paths. The resulting algorithm can be used as a tool for exploring the branching structure of RNA, especially for long sequences, and to examine branching structure dependence on energy model parameters. Full exposition is provided for the mathematical techniques used with the expectation that these techniques will prove useful in bioinformatics, computational biology, and additional extended applications.

## 1. Introduction

Computational and mathematical applications play a critical role in the analysis of the structure and function of biological molecules, including ribonucleic acid (RNA). RNA is an essential biological polymer with many roles including information transfer, regulation of gene expression, and catalysis of chemical reactions. The primary structure of an RNA molecule may be understood as a sequence of amino acids: arginine, urasil, guanine, and cytosine. As is standard, we frequently abbreviate these as A, U, G, and C, respectively. RNA molecules are single-stranded and may therefore interact with themselves, forming A–U, G–U, and G–C bonds. The secondary structure of an RNA molecule is a set of such bonds.

The determination of secondary structure is an important step to understanding an RNA molecule’s full shape and therefore its function [1,2]. Accordingly, secondary structure information is commonly used in tertiary structure prediction algorithms, see, e.g., [3–6]. Identifying the secondary structure of RNA is crucial to understanding its function and mechanism in a cell [7]. Thus, the structure of RNA is critical to the development of biological and pharmaceutical therapeutics. Biologists use inexpensive and expedient means to sequence RNA, but the experimental determination of structure is more difficult and time-consuming. Therefore, computational methods are the primary means to determine possible RNA secondary structures.

For decades, one of the main computational approaches for examining RNA structure and branching properties has been thermodynamic free energy minimization using Nearest Neighbor Thermodynamics Modeling (NNTM) [8–10]. This free energy is in turn used in algorithms to predict secondary structure given an RNA sequence, see, e.g., [11–13]. Under the NNTM, the free energy of a structure is computed as the sum of the free energy of its various substructures. Many common programs (e.g., mFold, RNAFold, RNA Structure, sFold, Vienna RNA, etc.) intake a single sequence to produce secondary structures based on NNTM energy minimizations performed via dynamic programming. Nearest neighbor parameter sets include both a set of rules, referred to as equations or features, and a set of parameter values used by the equations. Separate rules exist for predicting stabilities of helices, hairpin loops, small internal loops, large internal loops, bulge loops, multi-branch loops, and exterior loops. Other branching properties of interest include, but are not limited to, average ladder distance, maximum ladder distance, maximum branching degree, average contact distance, average branching degree, degree of branching at the exterior loop, number of multi-loops with n braches, etc. The online nearest neighbor database (NNDB) archives and stores complete nearest neighbor sets, including rules and corresponding parameter values [14].

A common challenge is inferring whether the predicted results of NNTM for a set of RNA structural features or branching properties are within expected dispersion thresholds for a given energy model. For example, is the number of hairpins more than 2–3 standard deviations greater than the expected mean for a given energy model? This challenge is particularly vexing if the sequence is relatively long (greater than 1000 nucleotides). If structural features or branching properties are determined to exceed expected energy model dispersion thresholds, it relays potential scientific and/or mechanistic insight. Continuing with our hairpin example, what if an NNTM model produces a result where the number of hairpins seems rather large for the given sequence length? If the number of hairpins exceeds the expected dispersion of the NNTM model, it might be inferred that the greater number of hairpins is evidence of natural selection.

The primary objective of the present study is to enable mathematical determination of the dispersion of RNA secondary structural features for a given sequence length. We present a Markov-based algorithm to provide samples of the branching structure under the NNTM and Gibbs distribution, but without reference to a particular sequence of nucleotides. The algorithm enables the determination of where the predicted feature or branching property for an actual sequence falls within this distribution, which in turn enables the determination of whether the predicted NNTM feature or branching property is within expected dispersion limits.

In particular, this work investigates RNA substructures called multi-loops, the places where three or more helices join. Though multi-loops are crucial to the overall shape of a secondary structure, the models used to predict them algorithmically do not produce accurate results [15]. This investigation builds on an existing model of RNA branching [16] and provides a theoretical grounding for a Markov chain which may be used to algorithmically investigate branching properties of secondary structure models. The investigational foundation is a model for RNA secondary structure developed by Hower and Heitsch [16], in which secondary structures are in bijection with plane trees and the minimal energy structures of the model have been previously characterized. The present study characterizes the full Gibbs distribution of possible structures. Notably, Bakhtin and Heitsch [17] analyzed a very similar model and determined degree sequence properties of the distribution of plane trees asymptotically. However, the present study utilizes a Markov chain-based sampling algorithm to investigate the Gibbs distribution in the finite case. A full explanation of the plane tree model as well as the derivation of the energy functions is provided in Section 2.1.

## 2. Methods

The methods are divided into an overview of the RNA secondary structure NNTM plane tree model and energy functions (Section 2.1) and an all-encompassing explanation of the mathematical preliminaries that lay the foundation for the derived results and corresponding algorithms (Section 2.2).

### 2.1. Derivation of Energy Functions

The energy function studied here is derived from the Nearest Neighbor Thermodynamic Model (NNTM). The numerical parameters from the NNTM can be found in the NNDB [14]. In calculating energy functions for the sequences, we consider thermodynamic parameter values published by Turner in 1989 [8], 1999 [9], and 2004 [10].

**Figure 1.**
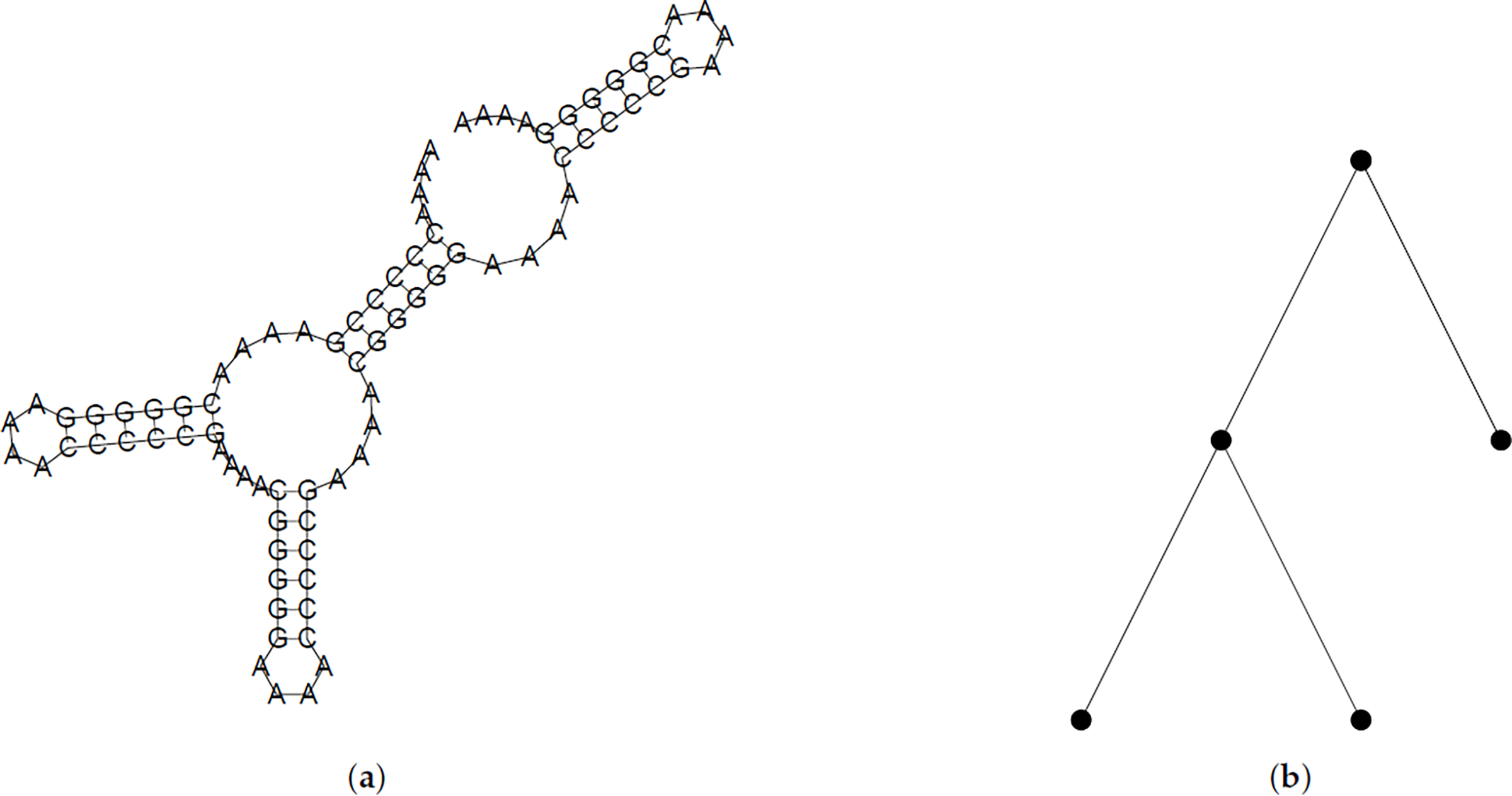
A ribonucleic acid (RNA) secondary structure for one of the combinatorial RNA sequences used in this work and its corresponding plane tree. The ordering of the edges in the plane tree is derived from the 3’ to 5’ ordering of the RNA sequence. Note that the exterior loop corresponds to the root of the plane tree. The diagram in (**a**) was generated by ViennaRNA [19]. (**a**) A maximally-paired secondary structure for *A*^4^(*C*^5^*GA*^4^*CG*^5^*A*^4^)^4^ has 4 helices; (**b**) The corresponding plane tree has 4 edges and encodes the branching pattern seen in the secondary structure.

The plane trees that we study in this paper come from two combinatorial RNA sequences, both of the form *A*^4^(*Y*^5^*ZA*^4^*YZ*^5^*A*^4^)*^n^*. The sequences of interest have (*Y*, *Z*) = (*C*, *G*) or (*Y*, *Z*) = (*G*, *C*). For both of these sequences, the set of maximally-paired secondary structures is in bijection with the set of plane trees of size *n* [18]. Figure 1 shows one example of a secondary structure and corresponding plane tree.

These specific combinatorial sequences are chosen because they allow for the study of the relationship between NNTM multiloop parameters and the branching behavior of secondary structures without interference from the energy contributions have specific base pairing combinations. In particular, the only places where the free energy differs between different secondary structures (for the same sequence) is in the type and number of multi-loops, the branching at the exterior loop, the number of hairpins, and the number of internal nodes. All of these energies directly relate to branching, not to specific base pairs. This simplification achieved by focusing only on multi-loops and branching both creates a model that is more amenable to theoretical analysis and speed computation.

Note that these secondary structures should not be considered representative of naturally occurring secondary structures. Instead, the only properties of interest in these structures are branching-related properties.

Three constants determine the free energy contribution of multiloops under NNTM, *a*, *b*, and *c*. The value of *a* encodes the energy penalty per multiloop. The constant *b* specifies the energy penalty per single-stranded nucleotide in a multiloop. The value of *c* gives the energy penalty for each helix branching from a multiloop.

In addition to the multiloop parameters *a*, *b*, *c* discussed above, we must account for the energy contributions of stacking base pairs, hairpins, interior loops, and dangling energy contributions. The energy of one helix is given by *h*. The energy associated with a hairpin is *f*, and the energy contribution of an interior loop is *i*. Finally, the parameter *g* encodes the dangling energy contributions. All of these values can be computed directly from the parameters found in the NNTM.

We wish to compute the energy of the structure corresponding to plane tree *t* having (down) degree sequence *d*_0_, *d*_1_, …, *d*_*n*−1_ and root degree *r*. Note that the down degree of a node *x* is equal to the number of children of *x*, and, in the down degree sequence, *d_i_* is the number of non-root nodes with exactly *i* children. The energy contribution of all hairpin loops will be *d*_0_ *f*, and similarly, the total energy of all interior loops will be *d*_1_*i*. For a multi-loop having down degree *j*, the energy contribution will be *a* + 4*b*(*j* + 1) + *c*(*j* + 1) + (*j* + 1)*g*, and so the contribution of all multi-loops is given by $\sum_{j=2}^{n}d_{j}(a+4b(j+1)+c(j+1)+g(j+1))$. Te root vertex of the tree corresponds to the exterior loop and has energy contribution *gr*. Finally, our structure has *n* helices, each with energy *h*. Summing all of these components gives the total energy.

(1) $$d_{0}f+d_{1}i+\sum_{j=2}^{n}d_{j}(a+4b(j+1)+c(j+1)+g(j+1))+nh+gr$$

(2) $$=\left(f-a-4b-c-g\right)d_{0}+\left(i-a-8b-2c-2g\right)d_{1}+\left(-4b-c\right)r+\left(a+8b+2c+h+2g\right)n,$$

where we have used the facts $\sum_{k=0}^{n-1}d_{k}=n$ and $\sum_{k=0}^{n-1}kd_{k}=n-r$.

Set *α* = *f* − *a* − 4*b* − *c* − *g*, *β* = *i* − *a* − 8*b* − 2*c* − 2*g*, *γ* = −4*b* − *c*, and *δ* = *a* + 8*b* + 2*c* + *h* + 2*g*. Then, the energy function is *αd*_0_ + *βd*_1_ +*γr* +*δn*. Since *n* will be fixed, we disregard the term *δn*, giving

(3) $$E(t)=\alpha d_{0}+\beta d_{1}+\gamma r.$$

Though we study these energy functions for arbitrary values of (*α*, *β*, *γ*), numerical values for both the input energy parameters from NNTM and the resulting energy function coefficients are given in Table 1.

### 2.2. Mathematical Preliminaries

Section 2.2 of this manuscript provides the necessary mathematical background, including a formal introduction of combinatorial objects and a review of the relevant Markov chain mixing results used to construct our resultant sampling Markov chain and corresponding mixing time proof in Section 3.

#### 2.2.1. Combinatorial Objects

A plane tree is a rooted, ordered tree. We will use $T_{n}$ to denote the set of plane trees with *n* edges. It is known that $\left|T_{n}\right|$ is given by the *n*th Catalan number $C_{n}=\frac{1}{n+1}\left(\begin{array}{c} 2n\\ n \end{array}\right)$. In a plane tree, a leaf is a node with down degree 0, and an internal node is a non-root node with down degree 1. For a given plane tree *t*, we will use *d*_0_(*t*) to denote the number of leaves and *d*_1_(*t*) to denote the number of internal nodes.

For a plane tree *t*, the energy of the tree is given by

(4) $$E(t)=\alpha d_{0}(t)+\beta d_{1}(t),$$

where *α* and *β* are real parameters of the energy function. Note that this function is a simplification of the model due to Hower and Heitsch [16] discussed in Section 2.1. Making this simplification effectively disregards the energy contribution of the exterior loop, which is small in comparison to the total energy of a structure, especially for the longer sequences that are of interest to us. Other authors have made similar simplifications, e.g., [17].

For our purposes, we consider *α* and *β* to be arbitrary but fixed. We will consider a Gibbs distribution **g** on the set $T_{n}$, where the weight of each tree *t* is given by

(5) $$g(t)=\frac{e^{-E(t)}}{Z},$$

where $Z=\sum_{y\in T_{n}}e^{-E(y)}$ is a normalizing constant.

A Motzkin path of length *n* is a lattice path from (0, 0) to (*n*, 0), which consists of steps along the vectors *U* = (1, 1), *H* = (1, 0), and *D* = (1, −1) and never crosses below the *x*-axis. We can also represent Motzkin paths as strings from the alphabet {*U*, *H*, *D*} where, in any prefix, the number of *U*s is greater than or equal to the number of *D*s. The number of Motzkin paths of length *n* is given by the Motzkin numbers *M_n_* where

(6) $$M_{n}=\sum_{k=0}^{\left\lfloor n/2\right\rfloor }\left(\begin{array}{c} n\\ 2k \end{array}\right)C_{k}.$$

Motzkin numbers and Motzkin paths have been well-studied in the combinatorics literature, see, e.g., [20–24].

A Dyck path is a Motzkin path with no *H* steps. It is easy to see that a Dyck path must have even length, so we will use $D_{n}$ to denote the set of Dyck paths on length 2*n*. It is well known that $\left|D_{n}\right|=C_{n}$ (see, e.g., [25]).

A 2-Motzkin path is a Motzkin path in which (1, 0) steps are given one of two distinguishable colors. Let $M_{m}^{2}$ be the set of all 2-Motzkin paths of length *m*. We can also represent 2-Motzkin paths as strings from the alphabet {*U*, *H*, *I*, *D*}, where, as before, the number of *D*s never exceeds the number of *U*s in any prefix. In a such a string *x*, we denote by |*x*|*_a_* the number of times the symbol *a* appears in *x*, where *a* ∈ {*U*, *H*, *I*, *D*}. Notice that we always have |*x*|*_U_* = |*x*|*_D_*. For any $x\in M_{n}^{2}$ and *k* ∈ {1, · · ·, *n*}, let *x*(*k*) denote the symbol at index *k* in the string representation of *x*. Additionally, the skeleton of a 2-Motzkin path *x* is the Dyck path of *U*s and *D*s which results from removing all *H*s and *I*s from *x*. We will denote the skeleton of *x* by *σ*(*x*).

#### 2.2.2. A Bijection Between $T_{n}$ and $M_{n-1}^{2}$

We will use the particular bijection $\Phi :{ℑ}_{n}\to M_{n-1}^{2}$ between plane trees and 2-Motzkin paths from Deutsch [26], which neatly encodes information about *d*_0_ and *d*_1_. For clarity, we will overview the bijection here.

For a given plane tree *t* with *n* edges, assign a label from the set {*U*, *H*, *I*, *D*} to each edge *e* according to the following rules:

- If *e* is the leftmost edge off a non-root node of down degree at least 2, assign the label *U*.
- If *e* is the rightmost edge off a non-root node of down degree at least 2, assign the label *D*.
- If *e* is the only edge off a non-root node of degree 1, assign the label *I*.
- If *e* is an edge off the root node, or if *e* is neither the leftmost nor the rightmost edge off its parent node, assign the label *H*.

**Figure 2.**
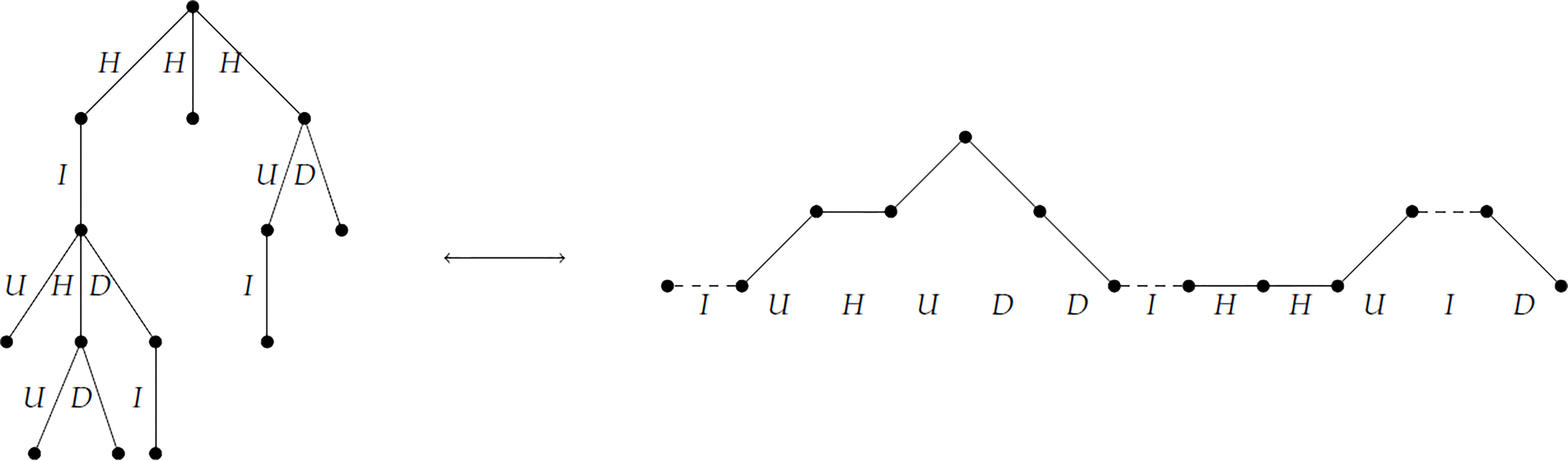
A plane tree with edges labeled according to the bijection Φ, along with its corresponding 2-Motzkin path.

Now, if we traverse *t* in a preorder reading off these labels, we get a 2-Motzkin path of length *n*. However, this path will always begin with *H*, so we define Φ(*t*) to be the 2-Motzkin path of length *n* − 1 after this initial *H* is removed. Figure 2 gives an example of this labeling process. From Deutsch, we know not only that Φ is a bijection, but also that if *x* = Φ(*t*) then |*x*|*_I_* = *d*_1_(*t*) and |*x*|*_U_* + |*x*|*_H_* + 1 = *d*_0_(*t*).

Using this bijection, it is natural to extend our energy function to 2-Motzkin paths. We define the energy of a 2-Motzkin path *x* to be

(7) $$E(x)=\alpha \left(|x{|}_{U}+|x{|}_{H}+1\right)+\beta |x{|}_{I},$$

and we extend our definition of the distribution **g** to $M_{n}^{2}$ accordingly. We note that, while this energy function does not capture all possible weightings on 2-Motzkin paths, it does capture all weightings possible under our simplification of the model due to Hower and Heitsch [16] after applying the bijection due to Deutsch [26].

#### 2.2.3. Markov Chains

A Markov chain M is a sequence of random variables *X*_0_, *X*_1_, *X*_2_, · · · taking values in a state space Ω subject to the condition that

(8) $$\mathrm{Pr}\left(X_{t+1}=y|X_{t}=x,X_{t-1}=s_{t-1},\cdots ,X_{0}=s_{0}\right)=\mathrm{Pr}\left(X_{t+1}=y|X_{t}=x\right).$$

All Markov chains that we consider will be implicitly time-homogeneous (meaning Pr(*X*_*t*+1_ = *y* | *X_t_* = *x*) does not depend on *t*) and finite (meaning |Ω| < ∞). The transition matrix of a time-homogeneous Markov chain is the matrix *P*: Ω × Ω → [0, 1] given by

(9) $$P(x,\;y)=\mathrm{Pr}\left(X_{t+1}=y|X_{t}=x\right).$$

It is easy to see that if *X*_0_ has distribution vector **x**, then *X_t_* has distribution vector *P^t^***x**.

A finite Markov chain with transition matrix *P* is said to be ergodic if it has the following two properties.

1. Irreducibility: For any *x*, *y* ∈ Ω, there is some integer t∈ℕ for which *P^t^*(*x*, *y*) > 0.
2. Aperiodicity: For any state *x* ∈ Ω, we have $\gcd\left\{t\in \mathbb{N}:P^{t}(x,\;x)>0\right\}=1$.

It is well-known that if M is ergodic, then there exists a unique distribution vector *π*, the stationary distribution, such that *Pπ* = *π*, and lim_*t*→∞_ *P^t^*(*x*, *y*) = *π*(*y*) for any states *x*, *y* ∈ Ω. Additionally, we call M reversible if for all states *x*, *y* ∈ Ω, we have *π*(*x*)*P*(*x*, *y*) = *π*(*y*)*P*(*y*, *x*).

For *ϵ* > 0, the mixing time *τ*(*ϵ*) of M is given by

(10) $$\tau (\epsilon )=\min\left\{t\in \mathbb{N}:\forall s\geq t,\;\underset{x\in \Omega }{\min}\left(\frac{1}{2}\sum_{y\in \Omega }\left|P^{s}(x,\;y)-\pi (y)\right|\right)<\epsilon \right\}.$$

Intuitively, the mixing time gives a measure of the number of steps required for M to get sufficiently close to its stationary distribution from any starting state.

Let M be a finite ergodic Markov chain over a state space Ω with transition matrix *P*. Let the eigenvalues of *P* be *λ*_0_, *λ*_1_, …, *λ*_|Ω|−1_ such that 1 = *λ*_0_ > |*λ*_1_| ≥ … ≥ |*λ*_|Ω|−1_|. The spectral gap of M is given by $\mathrm{Gap}(M)=1-\left|\lambda_{1}\right|$. As is standard, it will be convenient to denote the inverse of the spectral gap by relaxation time $\tau_{rel}(M):=1/\mathrm{Gap}(M)$.

Additionally, the spectral gap is given by the following functional definition [27].

(11) $$\mathrm{Gap}(M)=\underset{f}{\inf}\frac{\sum_{x,y\in \Omega }|f(x)\;-\;f(y){|}^{2}\pi (x)P(x,y)}{\sum_{x,y\in \Omega }|f(x)\;-\;f(y){|}^{2}\pi (x)\pi (y)},$$

where the infimum is taken over all non-constant functions f:Ω→ℝ. A direct consequence of this definition of the spectral gap is the following lemma.

##### Lemma 1.

*Let* $M_{1}$ *and* $M_{2}$ *be ergodic Markov chains over* Ω *with the same stationary distribution. Let P*_1_ *and P*_2_ *be the transition matrices of* $M_{1}$ *and* $M_{2}$ *respectively. If for all x*, *y* ∈ Ω *and for some constant c* > 0 *we have P*_1_(*x*, *y*) ≤ *cP*_2_(*x*, *y*), *then* $\mathrm{Gap}\left(M_{1}\right)\leq c\mathrm{Gap}\left(M_{2}\right)$.

Additionally, spectral gap is related to the mixing time by the following lemma [28].

##### Lemma 2.

*Let* M *be an ergodic Markov chain with state space* Ω, *and let λ*_0_, *λ*_1_, …, *λ*_|Ω|−1_ *be the eigenvalues of the transition matrix P as defined above. Then, for all ϵ* > 0 *and x* ∈ Ω, *we have*

(12) $$\frac{\left|\lambda_{1}\right|}{\mathrm{Gap}(M)}\log\left(\frac{1}{2\epsilon }\right)\leq \tau (\epsilon )\leq \frac{1}{\mathrm{Gap}(M)}\log\left(\frac{1}{\pi (x)\epsilon }\right).$$

We say that a Markov chain M, whose state space depends on a variable n∈ℕ, is rapidly mixing if *τ*(*ϵ*) is bounded above by some polynomial in *n* and log(*ϵ*^−1^). For the specific chains studied in this manuscript, we will show that τ(ϵ)(M) is bounded by a polynomial in *n* and log(*ϵ*^−1^) if and only if $\tau_{rel}(M)$ is bounded by a polynomial in *n* and *log*(*ϵ*^−1^). Our next lemma presents sufficient conditions.

##### Lemma 3.

*Let* M *be an ergodic Markov chain with state space* Ω *and let λ*_0_, *λ*_1_, …, *λ*_|Ω|−1_ *be the eigenvalues of its transition matrix. Let ϵ* > 0. *If τ*(*ϵ*) *is bounded by a polynomial in n and log*(*ϵ*^−1^), *then τ_rel_ is also bounded by a polynomial in n and* log(*ϵ*^−1^). *Further, suppose we have* log(1/*π*(*x*)) *bounded by some polynomial q*(*n*) *for all x* ∈ Ω. *Then*, $\tau_{rel}(M)$ *being bounded by a polynomial in n and* log(*ϵ*^−1^) *implies that τ*(*ϵ*) *is also bounded by some polynomial in n and* log(*ϵ*^−1^).

##### Proof.

Suppose that *τ*(*ϵ*) ≤ *p*(*n*, log(*ϵ*^−1^)), where *p* is a polynomial. Beginning with the left hand side of Lemma 2, note that

$$\frac{\left|\lambda_{1}\right|}{1\;-\;\left|\lambda_{1}\right|}\log\left(\frac{1}{2\epsilon }\right)=\left(\tau_{rel}(M)-1\right)\log\left(\frac{1}{2\epsilon }\right).$$

Then, applying Lemma 2 and the bound on *τ*(*ϵ*),

$$\tau_{\text{rel}}(M)\leq \frac{\tau (\epsilon )}{\log\left({\left(2\epsilon \right)}^{-1}\right)}+1\leq \frac{p\left(n,\;\log\left(\epsilon^{-1}\right)\right)}{\log\left({\left(2\epsilon \right)}^{-1}\right)}+1\leq p^{\prime }\left(n,\;\log\left(\epsilon^{-1}\right)\right),$$

where *p*′ is again a polynomial in *n* and log(*ϵ*^−1^).

Turning now to converse, suppose that we have *τ_rel_* ≤ *p*(*n*, log(*ϵ*^−1^)), for some polynomial *p*. Additionally suppose log(1/*π*(*x*)) ≤ *q*(*n*) for all *x* ∈ Ω, for some polynomial *q*.

Applying Lemma 2,

$$\tau (\epsilon )\leq \tau_{rel}(M)\log\left(\frac{1}{\pi (x)\epsilon }\right)\leq p\left(n,\;\log\left(\epsilon^{-1}\right)\right)\log\left(\epsilon^{-1}\right)q(n)\leq p^{\prime }\left(n,\;\log\left(\epsilon^{-1}\right)\right),$$

where *p*′ is some polynomial. □

#### 2.2.4. Coupling

A coupling of a Markov chain M on Ω is a chain ${\left(X_{t},\;Y_{t}\right)}_{t=0}^{\infty }$ on Ω × Ω for which the following properties hold.

1. Each chain ${\left(X_{t}\right)}_{t=0}^{\infty }$ and ${\left(Y_{t}\right)}_{t=0}^{\infty }$, when viewed in isolation, is a copy of M, given initial states *X*_0_ = *x* and *Y*_0_ = *y*.
2. Whenever *X_t_* = *Y_t_*, we have *X*_*t*+1_ = *Y*_*t*+1_.

Formally, the first item above requires that the joint distribution of (*X_t_*, *Y_t_*) (given (*X*_*t*−1_, *Y*_*t*−1_)) should satisfy the property that the marginal of *X_t_* (and also *Y_t_*) is consistent with the probability transitions of M. We define the coupling time *T* to be

(13) $$T=\underset{x,y\in \Omega }{\max}\;E\left[\min\left\{t:X_{t}=Y_{t}|X_{0}=x,\;Y_{0}=y\right\}\right].$$

The following lemma [29] is useful in bounding the coupling time *T*.

##### Lemma 4.

*Suppose that* ${\left(X_{t},\;Y_{t}\right)}_{t=0}^{\infty }$ *is a coupling of a Markov chain M. Let φ be an integer-valued distance function on* Ω × Ω *taking values in the range* [0, *B*], *and suppose that φ*(*x*, *y*) = 0 *if and only if x* = *y. Let φ*(*t*) = *φ*(*x_t_*, *y_t_*). *Suppose that the coupling satisfies E* (*φ*(*t* + 1) − *φ*(*t*)|*X_t_*, *Y_t_*) ≤ 0. *Additionally, suppose that whenever φ*(*t*) > 0, *E* |*φ*(*t* + 1) − *φ*(*t*)|^2^|*X_t_*, *Y_t_* ≥ *V. Then, the expected coupling time satisfies E* (*T*^x,y^) ≤ *φ*(0)(2*B* − *φ*(0))/*V.*

Coupling time and mixing time are then related by the following theorem [28].

##### Theorem 1.

*A Markov chain M with coupling time T has mixing time τ*(*ϵ*) *bounded by*

(14) $$\tau (\epsilon )\leq \left\lceil Te\log\epsilon^{-1}\right\rceil .$$

#### 2.2.5. Decomposition

We use two disjoint decomposition methods for bounding the spectral gap, one developed by Martin and Randall [30], and a very recent one given by Hermon and Salez [31], building on the work by Jerrum, Son, Tetali and Vigoda [32]. We use both theorems because, while the latter gives better bounds, the former has more relaxed conditions, which is necessary in one of our applications. The setup for both methods is the same.

Let M be an ergodic, reversible Markov chain over a state space Ω with transition matrix *P* and stationary distribution *π*. Suppose Ω can be partitioned into disjoint subsets Ω_1_, …, Ω*_m_*. For each *i* ∈ [*m*], let $M_{i}$ be the restriction of M to Ω*_i_*, which is obtained by rejecting any transition that would leave Ω*_i_*. Let *P_i_* be the transition matrix of $M_{i}$ Additionally, we define $\bar{M}$ to be the projection chain of M over the state space [*m*] as follows. Let the transition matrix $\bar{P}$ of $\bar{M}$ be given by

(15) $$\bar{P}(i,j)=\frac{1}{\pi \left(\Omega_{i}\right)}\sum_{\begin{array}{c} x\in \Omega_{i}\\ y\in \Omega_{j} \end{array}}\pi (x)P(x,y).$$

One can check that $\bar{M}$ is reversible and has stationary distribution

$$\bar{\pi }(i)=\pi \left(\Omega_{i}\right),$$

while each $M_{i}$ has stationary distribution

$$\pi_{i}(x)=\frac{\pi (x)}{\bar{\pi }(i)}.$$

With this notation, we have the following theorem by Martin and Randall [30].

##### Theorem 2.

Defining $M_{i}$ and $\bar{M}$ as above, we have

(16) $$\mathrm{Gap}(M)\geq \frac{1}{2}\mathrm{Gap}(\bar{M})\;\underset{i\in [m]}{\min}\mathrm{Gap}\left(M_{i}\right).$$

The theorem due to Hermon and Salez obtains better bounds if, for each pair (*i*, *j*) ∈ [*m*] × [*m*] with $\bar{P}(i,j)>0$, we can find an effective joint distribution (often referred to as a “coupling”) *κ_ij_* : Ω*_i_* × Ω*_j_* → [0, 1] of the distributions *π_i_* and *π_j_*. In other words, we must have

(17) $$\forall x\in \Omega_{i},\;\sum_{y\in \Omega_{j}}\kappa_{ij}(x,\;y)=\pi_{i}(x),$$

(18) $$\forall y\in \Omega_{j},\;\sum_{x\in \Omega_{i}}\kappa_{ij}(x,\;y)=\pi_{j}(y).$$

The quality of the joint distribution *κ* is defined as

(19) $$\chi :=\chi (\kappa ):=\min\left\{\frac{\pi (x)P(x,\;y)}{\bar{\pi }(i)\bar{P}(i,\;j)\kappa_{ij}(x,\;y)}\right\},$$

where the minimum is taken over all (*x*, *y*, *i*, *j*) with *x* ∈ Ω*_i_*, *y* ∈ Ω*_j_* for which $\bar{P}(i,\;j)>0$ and *κ_ij_*(*x*, *y*) > 0. Hermon and Salez [31] prove the following.

##### Theorem 3.

With P, $\bar{P}$, P_i_, and χ defined as above,

(20) $$\mathrm{Gap}(M)\geq \min\left\{\chi \mathrm{Gap}(\bar{M}),\underset{i\in [m]}{\min}\mathrm{Gap}\left(M_{i}\right)\right\}.$$

The utility of these decomposition theorems is that they allow us to break down a more complicated Markov chain into pieces that are easier to analyze. If we can show that the pieces rapidly mix, and the projection chain rapidly mixes, then we may conclude that the original chain rapidly mixes as well.

Additionally, to aid with the analysis of some projection chains, we will need another lemma from [30].

Let $M_{M}$ be the Markov chain on [*m*] with Metropolis transitions $P_{M}(i,\;j)=\frac{1}{2\Delta }\min\left\{1,\;\frac{\pi \left(\Omega_{j}\right)}{\pi \left(\Omega_{i}\right)}\right\}$ whenever $\bar{P}(i,\;j)>0$, where Δ is the maximum degree of vertices in the transition graph of $\bar{M}$. Let *∂_i_*(Ω*_j_*) = {*y* ∈ Ω*_j_* : ∃*x* ∈ Ω*_i_* with *P*(*x*, *y*) > 0}. Then we have the following

##### Lemma 5.

*With* $M_{M}$ *as defined above, suppose there exist constants a* > 0 *and b* > 0 *with*

1. *P*(*x*, *y*) ≥ *a for all x*, *y such that P*(*x*, *y*) > 0.
2. *π*(*∂_i_*(Ω*_j_*)) ≥ *bπ*(Ω*_j_*) *for all i*, *j with $\bar{P}(i,\;j)>0$.*

Then $\mathrm{Gap}(\bar{M})\geq ab\cdot \mathrm{Gap}\left(M_{M}\right)$.

In order to help analyze the mixing time of $M_{M}$, we will also require the following two lemmas. Note that Lemma 6 is used only in the proof of Lemma 7.

##### Lemma 6.

*Let* ${\left(a_{i}\right)}_{i=1}^{m}$ *be a log concave sequence, with a_i_* > 0 *for all* 1 ≤ *i* ≤ *m. Then,*

(21) $$\frac{a_{i+1}}{a_{i}}\geq \frac{a_{j+1}}{a_{j}}$$

*for all* 1 ≤ *i* ≤ *j* ≤ *m.*

##### Proof.

In order to use induction, we will slightly reframe the statement. We will prove

$$\frac{a_{i+1}}{a_{i}}\geq \frac{a_{i+1+k}}{a_{i+k}}$$

for all *i* + *k* ≤ *n*.

We now proceed by induction on *k*. The base case, *k* = 0, is trivial.

Now fix *l* > 0 and suppose that the induction hypothesis is true for *k* = *l* − 1, that is,

$$\frac{a_{i+1}}{a_{i}}\geq \frac{a_{i+l}}{a_{i+l-1}}.$$

By log concavity $a_{i+l}^{2}\geq a_{i+l-1}a_{i+l+1}$, or, equivalently,

$$\frac{a_{i+l}}{a_{i+l-1}}\geq \frac{a_{i+l+1}}{a_{i+l}}.$$

Therefore,

$$\frac{a_{i+1}}{a_{i}}\geq \frac{a_{i+l}}{a_{i+l-1}}\geq \frac{a_{i+l+1}}{a_{i+l}},$$

where the first inequality follows from the induction hypothesis, and the second inequality follows from log concavity. □

##### Lemma 7.

*Let π be a probability distribution on* [*m*]. *Let* M *be a Markov chain on* [*m*] *with the transition probabilities*

(22) $$P(i,\;j)=\{\begin{array}{ll} \frac{1}{4}\min\left\{1,\frac{\pi (j)}{\pi (i)}\right\} & \;\text{if}\;|i-j|=1\\ 0 & \;\text{if}\;|i-j|>1 \end{array}$$

*and the appropriate self-loop probabilities P*(*i*, *i*). *If π*(*i*) *is log-concave in i, then* M *has mixing time (and hence also relaxation time) O*(*m*^2^).

##### Proof.

We define a coupling (*X_t_*, *Y_t_*) on M as follows. If *X_t_* ≠ *Y_t_*, then at time step *t* + 1, flip a fair coin.

- If heads, set *Y*_*t*+1_ = *Y_t_*. Let *l* be either 1 or −1, each with probability 1/2. If possible, let *X*_*t*+1_ = *X_t_* + *l* with probability $\frac{1}{2}\;\min\left\{1,\;\frac{\pi \left(X_{t}+l\right)}{\pi \left(X_{t}\right)}\right\}$. Otherwise, let *X*_*t*+1_ = *X_t_*.
- If tails, set *X*_*t*+1_ = *X_t_*, and update *Y*_*t*+1_ the same way as we did for *X*_*t*+1_ in the previous case.

Now, suppose that for some *t* we have *X_t_* = *i* and *Y_t_* = *j* for *i* ≠ *j*. WLOG, assume that *i* < *j*. Let *φ*(*t*) = *φ*(*X_t_*, *Y_t_*) = *j* − *i*, and let Δ*φ*(*t*) = *φ*(*t*) − *φ*(*t* − 1). Note that we have two moves, with probabilities *P*(*i*, *i* − 1) and *P*(*j*, *j* + 1), which will increase the distance *φ* by 1 and similarly two moves, with probabilities *P*(*i*, *i* + 1) and *P*(*j*, *j* + 1), will decrease the distance by 1. Then we have

E(Δφ(t))=−P(i,i+1)+P(i,i−1)+P(j,j+1)−P(j,j−1).

By the log-concavity of *π*(*i*) and Lemma 6, we have *P*(*i*, *i* + 1) ≥ *P*(*j*, *j* + 1) and *P*(*i*, *i* − 1) ≤ *P*(*j*, *j* − 1). Therefore, the expected change in *φ*(*t*) is always non-positive. We also have

$$\begin{array}{l} E\left({\left(\Delta \varphi (t)\right)}^{2}|X_{t},\;Y_{t}\right)=P(j,\;j+1)+P(i,\;i+1)+P(j,\;j-1)+P(i,\;i-1)\\ =\frac{1}{4}\left(\min\left\{1,\;\frac{\pi (j\;+\;1)}{\pi (j)}\right\}+\min\left\{1,\;\frac{\pi (i\;+\;1)}{\pi (i)}\right\}+\min\left\{1,\;\frac{\pi (j\;-\;1)}{\pi (j)}\right\}+\min\left\{1,\;\frac{\pi (i\;-\;1)}{\pi (i)}\right\}\right). \end{array}$$

We claim that $E\left({\left(\Delta \varphi \right)}^{2}|X_{t},\;Y_{t}\right)\geq \frac{1}{4}$. Suppose, for contradiction, that the expectation is less than $\frac{1}{4}$. Then, for each of the minimum functions in the above expression, 1 must be the larger argument. Equivalently, *π*(*i* − 1) < *π*(*i*), *π*(*i*) > *π*(*i* + 1), *π*(*j* − 1) < *π*(*j*), and *π*(*j*) > *π*(*j* + 1).

Therefore, *π*(*i*) is not unimodal in *i* and is therefore also not log-concave in *i*, contradicting our hypothesis. Therefore we have $E\left({\left(\Delta \varphi \right)}^{2}|X_{t},\;Y_{t}\right)\geq \frac{1}{4}$, as desired. □

## 3. Results

Here we present the constructed Markov chain and corresponding algorithms devised for the sampling task and the proof of an upper bound on the relaxation time—that the chain mixes rapidly. Collectively, the results illustrate an analytical approach to calculate the dispersion of the secondary structure and corresponding branching properties of RNA based on the NNTM energy function minimization and without reference to a specific nucleotide sequence.

### 3.1. Our Markov Chain on $M_{m}^{2}$

We define a Markov chain $M=X_{0},\;X_{1},\;X_{2},\;\cdots$ on $M_{m}^{2}$ to sample 2-Motzkin paths as a representation of plane trees. Here, we use *m* = *n* − 1 to denote the length of the 2-Motzkin paths corresponding to plane trees with *n* edges.

We define each step of M as follows. First, pick a random element *l* uniformly from {1, 2, 3, 4}. Now choose *y* as follows.

- If *l* = 1, pick a random pair of consecutive symbols in *X_t_*, and call this pair *s*. If *s* is *UD* or *HH*, let *s*′ be either *UD* or *HH* with probabilities $\frac{1}{1+e^{-\alpha }}$ and $\frac{e^{-\alpha }}{1+e^{-\alpha }}$ respectively. Let *y* be the string *X_t_* with *s* replaced by *s*′. Otherwise, let *y* = *X_t_*.
- If *l* = 2, pick *i* uniformly from {1, · · ·, *m*}. If *X_t_*(*i*) is *H* or *I*, choose a symbol *c* to be either *H* or *I* with probabilities $\frac{e^{-\alpha }}{e^{-\alpha }+e^{-\beta }}$ and $\frac{e^{-\beta }}{e^{-\alpha }+e^{-\beta }}$ respectively. Let *y* be the 2-Motzkin path given by changing the symbol in *X_t_*(*j*) to *c*. Otherwise, we let *y* = *X_t_*.
- If *l* = 3, pick *i* and *j* each uniformly from {1, · · ·, *m*}. If each of *X_t_*(*i*) and *X_t_*(*j*) are either *U* or *D*, let *y* be the string *X_t_* with the symbols at indices *i* and *j* swapped. Otherwise, let *y* = *X_t_*.
- If *l* = 4, pick a random pair of consecutive symbols in *X_t_*, and call this pair *s*. If *s* is of the form *ab* or *ba* for some *a* ∈ {*U*, *D*} and *b* ∈ {*H*, *I*}, let *s*′ be the reverse of *s*, and let *y* be the string *X_t_* with *s* replaced by *s*′. Otherwise, let *y* = *X_t_*.

If *y* is a valid 2-Motzkin path, set *X*_*t*+1_ = *y* with probability $\frac{1}{2}$. Otherwise, set *X*_*t*+1_ = *X_t_*.

One can see that M is irreducible by noting that every path can be transformed to the path consisting of all *H*’s. To make this transformation, first use the *l* = 4 rule to move all *H*’s and *I*’s to the end of the path. If there are any *U*’s in the path, we must now have at least one consecutive pair *UD*. Use the *l* = 1 rule to convert the *UD* to a *HH*. From here we can repeat, again moving all *H*’s to the end and replacing *UD* with *HH*, until only *H*’s and *I*’s remain. Finally, we can use the *l* = 2 rule to convert all *I*’s to *H*’s. Since all of these steps can also be taken in reverse, this gives a procedure to move between two arbitrary paths, demonstrating irreducibility. We can also conclude that M is aperiodic, due to the existence of self-loops. Combined with irreducibility, this establishes that M is ergodic.

We claim that M is reversible with respect to the stationary distribution $\pi (x)=\frac{e^{-E(x)}}{Z}$, where $Z=\sum_{y\in M_{m}^{2}}e^{-E(y)}$. This can be easily verified by considering the four move types listed above. For example, for the first move type given above (transforming *UD* to *HH* and vice versa), let *x* and *y* be the states of interest. Suppose that *y* has the consecutive symbols *HH* where *x* contains *UD*. Then,

$$\begin{array}{l} \pi (x)P(x,\;y)=\frac{e^{-\alpha \left(|x{|}_{U}+|x{|}_{H}+1\right)-\beta |x{|}_{I}}}{Z}\cdot \frac{e^{-\alpha }}{1\;+\;e^{-\alpha }}\\ \;=\frac{e^{-\alpha \left(\left(|y{|}_{U}+1\right)+\left(|y{|}_{H}-2\right)+1\right)-\beta |y{|}_{I}}}{Z}\cdot \frac{e^{-\alpha }}{1\;+\;e^{-\alpha }}\\ \;=\frac{e^{-\alpha \left(|y{|}_{U}+|y{|}_{H}+1\right)-\beta |y{|}_{I}}}{Z}\cdot \frac{1}{1\;+\;e^{-\alpha }}\\ \;=\pi (y)P(y,\;x). \end{array}$$

One can verify that similar computations hold for the remaining three types of moves. Therefore, we conclude that the chain M has stationary distribution $\pi (x)=\frac{e^{-E(x)}}{Z}$.

The Markov chain M can be implemented in pseudocode as in Algorithm 1. Here, the Ber(*p*) function returns true with probability *p*, and false otherwise. We also use the addition of strings to denote concatenation.

Additionally, in order to convert the 2-Motzkin path *X_t_* into a plane tree, we use the algorithm in Algorithm 2, which assumes the existence of a Node object with children and parent attributes.

### 3.2. Mixing Time Results

Our main result is to prove the rapid mixing of the Markov chain defined in Section 3.1. An upper bound on the relaxation time is achieved by bounding the spectral gap from below. A spectral gap bound for the complex chain at hand is obtained through the use of multiple decomposition theorems, which give bounds on the spectral gap of the complex chain in terms of the spectral gaps of multiple simpler chains. The disjoint decomposition theorem due to Martin and Randall [30] provides a flexible approach to the decomposition of Markov chains. Very recent work by Hermon and Salez [31], building on the work of Jerrum, Son, Tetali, and Vigoda [32], proves a decomposition theorem with tighter bounds but stronger hypotheses.

Since this proof involves multiple decomposition steps, we provide an overview here. The primary tools used in this proof are the two decomposition theorems presented in Section 2.2.5. We first partition the state space of all 2-Motzkin paths by the number of *U*s in the path. The projection chain from this first decomposition is linear and is proved to be rapidly mixing using a result of Martin and Randall [30] (Lemma 8). Each of the restriction chains are decomposed again, this time by the pattern of *H* and *I* symbols. The projection chains for this second decomposition are shown to be rapidly mixing by coupling (Lemma 9). The restriction chains are decomposed a third time, this time according to the skeleton of *U* and *D* steps. The projection chains for this third decomposition are shown to be rapidly mixing by comparison to the classic mountain valley moves chain on Dyck paths (Lemma 10). This last set of restriction chains are found to be rapidly mixing by isomorphism to the chain consisting of adjacent transpositions on binary strings (Lemma 11). Finally, starting from the most restricted chains, we use the decomposition theorems to obtain a bound on the spectral gap of the original chain (Theorem 4).

**Algorithm 1:** The main Markov chain algorithm. This pseudocode calculates *X_t_* given *X*_0_.

**Require:** *X*_0_ is a valid 2-Motzkin path of length *m*.
*x* ← *X*_0_
**for** *s* = 1 → *t* **do**
*y ← x*
*l* ← randInt(1, 4)
**if** *l* = 1 **then**
*i* ← randInt(1, *m* − 1)
**if** *x*[*i*: *i* + 1] = *UD* **and** $\mathrm{Ber}\left(\frac{e^{-\alpha }}{2\left(1+e^{-\alpha }\right)}\right)$ **then**
*y*[*i*: *i* + 1] ← *HH*
**else if** *x*[*i*: *i* + 1] = *HH* **and** $\mathrm{Ber}\left(\frac{1}{2\left(1+e^{-\alpha }\right)}\right)$ **then**
*y*[*i*: *i* + 1] ← *UD*
**else if** *l* = 2 **then**
*i* ← randInt(1, *m*)
**if** *x*[*i*] = *I* **and** $\mathrm{Ber}\left(\frac{e^{-\alpha }}{2\left(e^{-\alpha }+e^{-\beta }\right)}\right)$ **then**
*y*[*i*] ← *H*
**else if** *x*(*i*) = *H* **and** $\text{Ber}\left(\frac{e^{-\beta }}{2\left(e^{-\alpha }+e^{-\beta }\right)}\right)$ **then**
*y*[*i*] ← *I*
**else if** *l* = 3 **then**
*i* ← randInt(1, *m*)
*j* ← randInt(1, *m*)
**if** (*x*[*i*] ∈ {*U, D*} **and** *x*[*j*] ∈ {*U, D*}) **and** $\mathrm{Ber}\left(\frac{1}{2}\right)$ **then**
*y*[*i*] ← *x*[*j*]
*y*[*j*] ← *x*[*i*]
**if** *y* is not a valid 2-Motzkin path **then**
*y* ← *x*
**else if** *l* = 4 **then**
*i* ← randInt(1, *m* − 1)
**if** (*x*[*i*] ∈ {*U, D*} **and** *x*[*j* + 1] ∈ {*H, I*}) **or** (*x*[*i*] ∈ {*H, I*} **and** *x*[*j + 1*] ∈ {*U, D*}) **and** $\mathrm{Ber}\left(\frac{1}{2}\right)$ **then**
*y*[*i*: *i* + 1] ← *x*[*j* + 1] + *x*[*j*]
*x* ← *y*
**return** *x*

We now proceed with a formal presentation. We will use a series of decompositions of M. We will first decompose our state space $M_{m}^{2}$ into $S_{0},\cdots ,S_{\left\lfloor m/2\right\rfloor }$, where

$$S_{k}=\left\{x\in M_{m}^{2}:|x{|}_{U}=k\right\}.$$

Let $M_{k}$ denote the Markov chain M restricted to the set *S_k_*, and let $\bar{M}$ be the projection chain over this decomposition as outlined for Theorem 2.

Additionally, we will decompose each *S_k_* into the sets {*T*_*k*,*q*_ : *q* ∈ (*H* + *I*)^*m*−2*k*^}, where (*H* + *I*)^*m*−2*k*^ denotes the set of strings with length *m* − 2*k* from the alphabet {*H*, *I*}. We define *T*_*k*,*q*_ to be the set of 2-Motzkin paths *x* ∈ *S_k_* such that the substring of *H* and *I* symbols in *x* is *q*. Let $M_{k,q}$ denote the chain $M_{k}$ restricted to *T*_*k*,*q*_, and let ${\bar{M}}_{k}$ be the projection chain of $M_{k}$ over this decomposition.

Finally, we decompose each *T*_*k*,*q*_ into the partition $\left\{U_{k,q,s}:s\in D_{k}\right\}$ based on the skeletons of the 2-Motzkin paths. For each $s\in D_{k}$, we define

$$U_{k,q,s}=\left\{x\in T_{k,q}|\sigma (x)=s\right\}.$$

**Figure 3.**
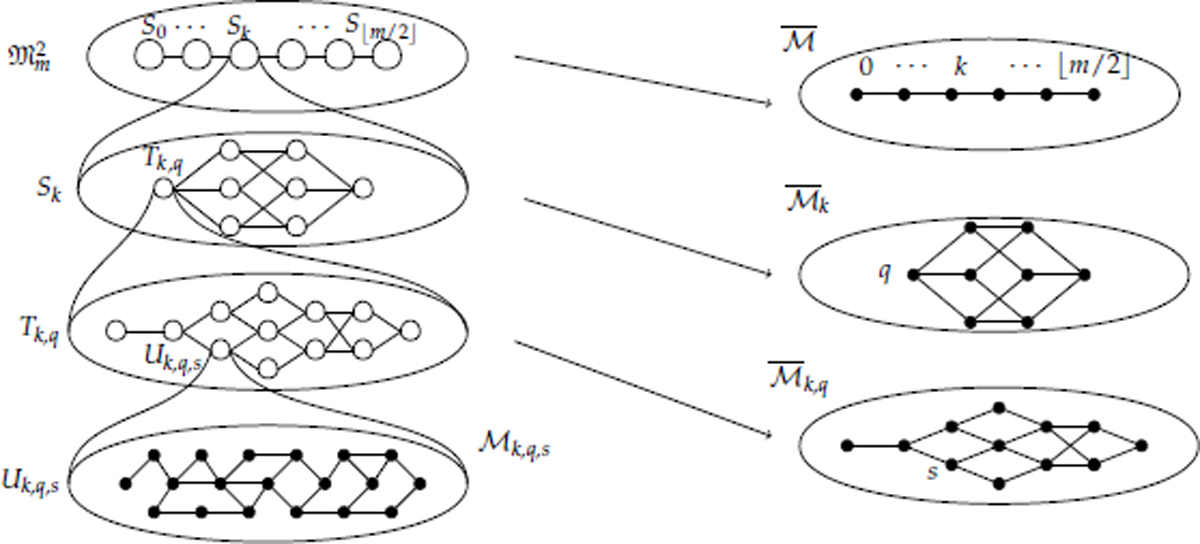
The four level decomposition of $M_{m}^{2}$ (**left**), and the projection chains corresponding to each decomposition (**right**).

As before, we let $M_{k,q,s}$ be the Markov chain $M_{k,q}$ restricted to *U*_*k*,*q*,_*_s_*, and let ${\bar{M}}_{k,q}$ be the appropriate projection chain. For clarity, this four-level decomposition is summarized in Figure 3.

**Algorithm 2:** Algorithm to convert a sampled 2-Motzkin path to a plan tree. The pseudocode calculates φ^−1^(*x*).

**Require:** *x* is a valid 2-Motzkin path of length *m*.
root ← new Node()
// *u* will be where a new node will be added for an *H* or *D* symbol
*u* ← root
// *v* will be always the last node added
*v* ← new Node()
// the stack will keep track of previous values of *u*
stack = new Stack()
root.children.append(*v*)
**for** *i* = 1 → *m* **do**
node ← new Node()
**if** *x*[*i*] = *U* **then**
*v*.children.append(node)
stack.push(*u*)
*u* ← *v*
**else if** *x*[*i*] = *I* **then**
*v*.children.append(node)
**else if** *x*[*i*] = *H* **then**
*u*.children.append(node)
**else if** *x*[*i*] = *D* **then**
*u*.children.append(node)
*u* ← stack.pop()
*v* ← node
**return** root

#### Lemma 8.

$\bar{M}$ has relaxation time $\tau_{rel}(\bar{M})=O\left(m^{4}\right)$.

#### Proof.

The chain $\bar{M}$ is a linear chain with states *k* in {0, …, ⌊*m*/2⌋}, and with stationary distribution

$$\begin{array}{l} \bar{\pi }(k)=\pi \left(S_{k}\right)=\frac{C_{k}}{Z_{m}}\cdot \sum_{i=0}^{m-2k}\left(\begin{array}{c} m\\ 2k \end{array}\right)\left(\begin{array}{c} m-2k\\ i \end{array}\right)e^{-\alpha (k+i+1)-\beta (m-2k-i)}\\ \;=\frac{e^{-\alpha (k+1)}}{Z_{m}}\left(\begin{array}{l} m\\ 2k \end{array}\right)C_{k}\cdot {\left(e^{-\alpha }+e^{-\beta }\right)}^{m-2k}, \end{array}$$

where $\bar{\pi }$ is defined as in Section 2.2.5. Notice that transitions in M which move between the *S_k_* sets are those which change a *HH* substring into a *UD* or *DU* substring, or vise versa. Thus, the transitions in $\bar{M}$ only increase or decrease *k* by at most 1. We seek to apply Lemma 5. To choose *a*, notice that for *x* ∈ *S_k_* and *y* ∈ *S*_*k*±1_ with *P*(*x*, *y*) > 0, we have

$$P(x,y)=\frac{1}{4(m\;-\;1)}\frac{1}{1\;+\;e^{\alpha }}\;\text{or}\;P(x,\;y)=\frac{1}{4(m\;-\;1)}\frac{1}{1\;+\;e^{-\alpha }}.$$

Note that the factor 1/4 comes from the choice *l* = 4, and the factor 1/(*m* − 1) comes from the fact that there are *m* − 1 adjacent pairs to pick from. Then,

$$P(x,\;y)\geq \frac{1}{4(m\;-\;1)(1\;+\;e^{-|\alpha |)}}.$$

Thus, we pick $a=\frac{1}{4(m-1)\left(1+e^{-|\alpha |}\right)}$.

To pick *b*, we let

$$\partial_{-}\left(S_{k}\right)=\left\{y\in S_{k}:\exists x\in S_{k-1},\;P(x,\;y)>0\right\}$$

for *k* ∈ {1, · · ·, ⌊*m*/2⌋}, and we let

$$\partial_{+}\left(S_{k}\right)=\left\{y\in S_{k}:\exists x\in S_{k+1},\;P(x,\;y)>0\right\}$$

for *k* ∈ {0, · · ·, ⌊*m*/2⌋ − 1}.

Additionally, let *A_k_* for *k* ∈ {1, · · ·, ⌊*m*/2⌋} be the subset of *S_k_* consisting of the 2-Motzkin paths in which the first *D* symbol appears immediately after a *U*. Let *B_k_* for *k* ∈ {0, · · ·, ⌊*m*/2⌋ − 1} be the subset of *S_k_* consisting of the 2-Motzkin paths in which a pair of adjacent *H* symbols occur before all other *H* or *I* symbols. It is easy to see that *A_k_* ⊂ *∂*_−_(*S_k_*) and *B_k_* ⊂ *∂*_+_(*S_k_*). We have

$$\pi \left(A_{k}\right)=\frac{C_{k}e^{-\alpha (k+1)}}{Z_{m}}\left(\begin{array}{l} m-1\\ 2k-1 \end{array}\right){\left(e^{-\alpha }+e^{-\beta }\right)}^{m-2k},$$

as there are *C_k_* ways to arrange the *U* and *D* symbols and $\left(\begin{array}{l} m-1\\ 2k-1 \end{array}\right)$ ways to insert *m* − 2*k H* or *I* symbols (treating *H* and *I* as being identical for now) without placing anything between the first *D* and the *U* immediately before it. The energy contribution of the *U* and *D* symbols is given by *e*^−*α*(*k*+1)^, and the energy contribution of the *H* and *I* symbols is (*e*^−*α*^ + *e*^−*β*^)^*m*−2*k*^. The required normalizing constant is *Z_n_*. Similarly, we also get

$$\pi \left(B_{k}\right)=\frac{C_{k}e^{-\alpha (k+3)}e^{-2\beta }}{Z_{m}}\left(\begin{array}{c} m-1\\ 2k \end{array}\right){\left(e^{-\alpha }+e^{-\beta }\right)}^{m-2k-2}$$

because there are *C_k_* ways to arrange the *U* and *D* symbols and $\left(\begin{array}{c} m-1\\ 2k \end{array}\right)$ ways insert *m* − 2*k* − 1 *H* or *I* symbols (treating the initial pair of *H*’s as a single symbol gives us only *m* − 2*k* − 1 symbols to insert). The energy contribution of the *U*’s, *D*’s, and the initial two *H*’s is given by $e^{-\alpha (k+3)}e^{-2\beta }$, and the energy contribution of the remaining *H*’s and *I*’s is (*e*^−*α*^ + *e*^−*β*^)^*m*−2*k*−2^. Finally, *Z_m_* is again a normalizing constant.

Hence combining these two results, we have

$$\frac{\pi \left(\partial_{-}\left(S_{k}\right)\right)}{\pi \left(S_{k}\right)}\geq \frac{\pi \left(A_{k}\right)}{\pi \left(S_{k}\right)}=\frac{2k}{m}$$

and

$$\frac{\pi \left(\partial_{+}\left(S_{k}\right)\right)}{\pi \left(S_{k}\right)}\geq \frac{\pi \left(B_{k}\right)}{\pi \left(S_{k}\right)}=\frac{m\;-\;2k}{m}{\left(\frac{e^{-\alpha }e^{-\beta }}{e^{-\alpha }\;+\;e^{-\beta }}\right)}^{2}.$$

Thus, we may let $b=\frac{1}{m}{\left(\frac{e^{-\alpha }e^{-\beta }}{e^{-\alpha }+e^{-\beta }}\right)}^{2}$.

Applying Lemma 5, we get that $\mathrm{Gap}(\bar{M})\geq \frac{\mathrm{Gap}\left(M_{M}\right)}{O\left(m^{2}\right)}$. Additionally, one can check that $\bar{\pi }(i)$ is log concave in *i*. Hence, using Lemma 7, we get $\tau_{rel}\left(M_{M}\right)=O\left(m^{2}\right)$, and in turn $\tau_{rel}(\bar{M})=O\left(m^{4}\right)$, as claimed. □

#### Lemma 9.

${\bar{M}}_{k}$ has mixing time $\tau \left({\bar{M}}_{k}\right)=O(m\;\log\;m)$, for all k.

#### Proof.

Notice that ${\bar{M}}_{k}$ appears as a chain with states *q* in the set *Q* = (*H* + *I*)^*m*−2*k*^. Additionally, transitions in ${\bar{M}}_{k}$ only occur between strings in *Q* that differ at only one index. The stationary distribution of ${\bar{M}}_{k}$ is given by ${\bar{\pi }}_{k}(q)\propto e^{(\beta -\alpha )|q{|}_{H}}$, where we have intentionally used the constant of proportionality to remove all dependence on *k*, which we consider, in this context, to be fixed.

Additionally, for *q*_1_, *q*_2_ ∈ *Q* which differ at exactly one index, we have the transition probability

$${\bar{P}}_{k}\left(q_{1},q_{2}\right)=\{\begin{array}{ll} \frac{(m-2k)e^{-\alpha }}{4m\left(e^{-\alpha }+e^{-\beta }\right)} & \;\text{if}\;{\left|q_{2}\right|}_{H}={\left|q_{1}\right|}_{H}+1\\ \frac{(m-2k)e^{-\beta }}{4m\left(e^{-\alpha }+e^{-\beta }\right)} & \;\text{if}\;{\left|q_{2}\right|}_{H}={\left|q_{1}\right|}_{H}-1 \end{array}.$$

We may show that ${\bar{M}}_{k}$ rapidly mixes by a simple coupling argument. Let ${\left(X_{t},\;Y_{t}\right)}_{t=0}^{\infty }$ be our coupled Markov chain on *Q* × *Q*. We define one step in this coupled chain as follows.

1. With probability $1-\frac{m-2k}{4m}$, set (*X*_*t*+1_, *Y*_*t*+1_) = (*X_t_*, *Y_t_*).
2. Otherwise, pick a random index *j* ∈ [*m* − 2*k*]. Let *a* ∈ {*H*, *I*} be a random symbol such that $\mathrm{Pr}(a=H)=\frac{e^{-\alpha }}{e^{-\alpha }+e^{-\beta }}$ and $\mathrm{Pr}(a=I)=\frac{e^{-\beta }}{e^{-\alpha }+e^{-\beta }}$. Now let *X*_*t*+1_ and *Y*_*t*+1_ be *X_t_* and *Y_t_* respectively, each with the *j*th symbol changed to *a*.

One can check that each of (*X_t_*)*_t_* and (*Y_t_*)*_t_* are indeed copies of ${\bar{M}}_{k}$. Additionally, notice that we will have *X_t_* = *Y_t_* after all *m* − 2*k* possible indices *j* have been updated. By the Coupon Collector Theorem, we have the coupling time of this chain to be $T_{{\bar{M}}_{k}}=\frac{4m}{m-2k}\cdot O((m-2k)\log(m-2k))=O(m\log m)$. Thus, using Theorem 1, we have the mixing time (and the relaxation time) also *O*(*m* log *m*). □

#### Lemma 10.

${\bar{M}}_{k,q}$ *has relaxation time* $\tau_{rel}\left(\bar{M_{k,q}}\right)=O\left(m^{2}\right)$, *for all pairs* (*k*, *q*).

#### Proof.

Notice that all *x* ∈ *T*_*k*,*q*_ have equal energy, and that $\left|U_{k,q,s}\right|=\left(\begin{array}{c} m\\ 2k \end{array}\right)$ for all *s*. Thus, ${\bar{M}}_{k,q}$ has a uniform stationary distribution. If we represent each set *U*_*k*,*q*,*s*_ by the Dyck path *s*, we can think of ${\bar{M}}_{k,q}$ as a chain over $D_{k}$. Since all the transitions in $M_{k,q}$ that move between the *U*_*k*,*q*,*s*_ sets are moves that exchange the positions of a *U* and a *D*, the transitions in ${\bar{M}}_{k,q}$ are simply the moves on elements of $D_{k}$ which exchange a *U* with a *D*. We call these moves on the elements of $D_{k}$, transposition moves.

For each $s_{1},\;s_{2}\in D_{k}$ that differ by a transposition move, the transition probabilities in our projection chain are given by

$$\begin{array}{l} {\bar{P}}_{k,q}\left(s_{1},\;s_{2}\right)=\frac{1}{\pi \left(U_{k,q,s_{1}}\right)}\sum_{\begin{array}{c} x\in U_{k,q,s_{1}}\\ y\in U_{k,q,s_{2}} \end{array}}\pi (x)P(x,y)=\frac{1}{\left|U_{k,q,s}\right|}\sum_{\begin{array}{c} x\in U_{k,q,s_{1}}\\ y\in U_{k,q,s_{2}} \end{array}}P(x,y)\\ \;=\frac{1}{\left(\begin{array}{c} m\\ 2k \end{array}\right)}\sum_{\begin{array}{c} x,y\\ P(x,y)>0 \end{array}}\frac{1}{4m^{2}}=\frac{1}{4m^{2}}. \end{array}$$

The last equality above relies on counting the number of terms in the sum. Notice that for each $x\in U_{k,q,s_{1}}$, there is a unique $y\in U_{k,q,s_{2}}$ for which *P*(*x*, *y*) > 0. Therefore, the number of terms is simply $\left|U_{k,q,s_{1}}\right|=\left(\begin{array}{c} m\\ 2k \end{array}\right)$. Compare this chain to the traditional mountain valley Markov chain on $D_{k}$, which we will denote by $M^{\prime }$. The transition probabilities of $M^{\prime }$ are given by $P^{\prime }\left(s_{1},\;s_{2}\right)=\frac{1}{k^{2}}$ for each pair (*s*_1_, *s*_2_) which differ by a mountain-valley move. It is known from Cohen [33] that $\mathrm{Gap}\left(M^{\prime }\right)=\frac{1}{O\left(k^{2}\right)}$. Thus, applying Lemma 1 to ${\bar{M}}_{k,q}$ and $M^{\prime }$, we see that $\mathrm{Gap}\left({\bar{M}}_{k,q}\right)=\frac{1}{O\left(m^{2}\right)}$. □

#### Lemma 11.

$M_{k,q,s}$ *has relaxation time* $\tau_{rel}\left(M_{k,q,s}\right)=O\left(m^{3}\right)$, *for all valid triples* (*k*, *q*, *s*).

#### Proof.

Notice that transitions in $M_{k,q,s}$ consist only of moves which involve swapping an *H* or an *I* with an adjacent *U* or *D*. Additionally, all 2-Motzkin paths in *U*_*k*,*q*,*s*_ have equal energy, so for all *x*, *y* ∈ *U*_*k*,*q*,*s*_ such that *P*(*x*, *y*) > 0, we have $P(x,\;y)=\frac{1}{8(m-1)}$.

To determine the mixing time of $M_{k,q,s}$, consider an isomorphic chain. Let *U*′ be the set of all binary strings of length *m* with 2*k* zeros and *m* − 2*k* ones. Let $M^{\prime }$ be the Markov chain on *U*′ where each step does nothing with probability 7/8 and swaps a random pair of adjacent (potentially identical) digits with probability 1/8. From Wilson [34], we know that the spectral gap of $M^{\prime }$ is $\frac{1}{O\left(m^{3}\right)}$. □

Finally, we can combine our bounds on the spectral gaps of all of these chains to prove our main result.

#### Theorem 4.

The Markov chain M has relaxation time $\tau_{rel}(M)=O\left(m^{7}\right)$, for all α,β∈ℝ.

#### Proof.

We use Lemmas 11 and 10 with Theorem 3 to obtain a bound on $\mathrm{Gap}\left(M_{k,q}\right)$. We define a coupling $\kappa_{s_{1},s_{2}}$ for each pair $\left(s_{1},\;s_{2}\right)\in D_{k}\times D_{k}$ with ${\bar{P}}_{k,q}\left(s_{1},\;s_{2}\right)>0$. For each such pair, notice that the set of pairs $(x,y)\in U_{k,q,s_{1}}\times U_{k,q,s_{2}}$ with *P*(*x*, *y*) > 0 is a perfect matching. Thus, we may set

$$\kappa_{s_{1},s_{2}}(x,y)=\{\begin{array}{ll} \frac{1}{\left(\begin{array}{l} m\\ 2k \end{array}\right)} & \;\text{if}\;P(x,y)>0\\ 0 & P(x,y)=0 \end{array}.$$

To compute *χ*, we begin by observing *π*(*x*) = *π*(*y*) for all $x,y\in M_{k,q}$. Note also $\left|U_{k,q,s}\right|=\left(\begin{array}{l} m\\ 2k \end{array}\right)$ for all skeletons *s* of length 2*k*. Before computing *χ*, we start by finding $\bar{P}\left(s_{1},\;s_{2}\right)$.


$$\begin{array}{l} \bar{P}\left(s_{1},\;s_{2}\right)=\frac{1}{\pi \left(U_{k,q,s_{1}}\right)}\sum_{x\in U_{k,q,s_{1}},y\in U_{k,q,s_{2}}}\pi (x)P(x,\;y)\\ \;=\frac{1}{\pi \left(U_{k,q,s_{1}}\right)}\sum_{x\in U_{k,q,s_{1}},y\in U_{k,q,s_{2}}}\frac{\pi (x)}{\frac{1}{4}\left(\begin{array}{c} m\\ 2 \end{array}\right)}\\ \;=\frac{1}{\pi \left(U_{k,q,s_{1}}\right)}\left|U_{k,q,s_{1}}\right|\frac{4\pi (x)}{\left(\begin{array}{c} m\\ 2 \end{array}\right)}\\ \;=\frac{4}{\left(\begin{array}{c} m\\ 2 \end{array}\right)}. \end{array}$$

We now proceed with the calculation of *χ*. Recall that the minimum is taken over all tuples *x*, *y*, *s*_1_, *s*_2_ where $\bar{P}\left(s_{1},\;s_{2}\right)>0$ and $\kappa_{0_{1},s_{2}}(x,\;y)>0$.


$$\begin{array}{l} \chi =\min\left\{\frac{\pi (x)P(x,\;y)}{\bar{\pi }\left(s_{1}\right)\bar{P}\left(s_{1},\;s_{2}\right)\kappa_{s_{1},s_{2}}(x,\;y)}\right\}\\ \;=\min\left\{\frac{\pi (x)\frac{4}{\left(\begin{array}{c} m\\ 2 \end{array}\right)}}{\pi \left(U_{k,q,s_{1}}\right)\frac{4}{\left(\begin{array}{c} m\\ 2 \end{array}\right)}\frac{1}{\left(\begin{array}{c} m\\ 2k \end{array}\right)}}\right\}\\ \;=\frac{\left(\begin{array}{l} m\\ 2k \end{array}\right)}{\left(\begin{array}{c} m\\ 2k \end{array}\right)}=1. \end{array}$$

Theorem 3 then gives

$$\begin{array}{l} \mathrm{Gap}\left(M_{k,q}\right)\geq \min\left\{\chi \mathrm{Gap}\left({\bar{M}}_{k,q}\right),\;\underset{s}{\min}\mathrm{Gap}\left(M_{k,q,s}\right)\right\}\\ \;\min\left\{\frac{1}{O\left(m^{2}\right)},\frac{1}{O\left(m^{3}\right)}\right\}\\ \;=\frac{1}{O\left(m^{3}\right)}. \end{array}$$

Similarly, we define a coupling $\kappa_{q_{1},q_{2}}$ for each pair (*q*_1_, *q*_2_) ∈ (*H* + *I*)^*m*−2*k*^ × (*H* + *I*)^*m*−2*k*^ with ${\bar{P}}_{k}\left(q_{1},\;q_{2}\right)>0$ to apply Theorem 3 to ${\bar{M}}_{k}$. Notice that once again, the set of pairs $(x,\;y)\in T_{k,q_{1}}\times T_{k,q_{2}}$ for which *P*(*x*, *y*) > 0 forms a perfect matching. Thus, we take

$$\kappa_{q_{1},q_{2}}(x,\;y)=\{\begin{array}{ll} \frac{1}{\left(\begin{array}{l} m\\ 2k \end{array}\right)C_{k}} & \;\text{if}\;P(x,\;y)>0\\ 0 & P(x,\;y)=0 \end{array}.$$

To compute *χ* for this coupling, we again begin with a few preliminary computations. In all of the following, let $x\in T_{k,q_{1}}$, $y\in T_{k,q_{2}}$ with $\bar{P}\left(q_{1},\;q_{2}\right)>0$. Note that *q*_1_ and *q*_2_ have the same length and differ at only one index. We will show the computations for the case where *q*_1_ has a *I* where *q*_2_ has a *H*. The computations for the other case are nearly identical.

Note that $P(x,\;y)=\frac{e^{-\alpha }}{e^{-\alpha }+a^{-\beta }}$. Note also

$$\bar{\pi }\left(q_{1}\right)=\pi \left(T_{k,q_{1}}\right)=\pi (x)\left|T_{k,q_{1}}\right|=\pi (x)C_{k}\left(\begin{array}{c} m\\ 2k \end{array}\right)$$

and

$$\begin{array}{l} \bar{P}\left(q_{1},\;q_{2}\right)=\frac{1}{\pi \left(T_{k,q_{1}}\right)}\sum_{x^{\prime }\in T_{k,q_{1}},y^{\prime }\in T_{k,q_{2}}}P\left(x^{\prime },\;y^{\prime }\right)\\ \;=\frac{1}{\left|T_{s,q_{1}}\right|}\cdot \frac{e^{-\alpha }}{e^{-\alpha }\;+\;e^{-\beta }}\left|T_{s,q_{1}}\right|\\ \;=\frac{e^{-\alpha }}{e^{-\alpha }\;+\;e^{-\beta }}. \end{array}$$

Now we can compute

$$\begin{array}{l} \chi =\min\left\{\frac{\pi (x)P(x,y)}{\bar{\pi }\left(q_{1}\right)\bar{P}}\left(q_{1},q_{2}\right)\kappa_{q_{1},q_{2}}(x,y)\right\}\\ \;=\min\left\{\frac{\pi (x)\frac{e^{-\alpha }}{e^{-\alpha }+e^{-\beta }}}{\pi (x)C_{k}\left(\begin{array}{l} m\\ 2k \end{array}\right)\frac{e^{-\alpha }}{e^{-\alpha }+e^{-\beta }}\cdot \frac{1}{C_{k}\left({}_{2k}^{m}\right)}}\right\}\\ \;=1. \end{array}$$

Applying Theorem 3 then gives

$$\begin{array}{l} \mathrm{Gap}\left(M_{k}\right)\geq \min\left\{\chi \mathrm{Gap}\left(\bar{M_{k}}\right),\underset{q}{\min}\mathrm{Gap}\left(M_{k,q}\right)\right\}\\ \;=\min\left\{\frac{1}{O(m\log m)},\frac{1}{O\left(m^{3}\right)}\right\}\\ \;=\frac{1}{O\left(m^{3}\right)}. \end{array}$$

Unfortunately, we have not been able to find a useful coupling for $\bar{M}$, so for the last step of our decomposition, we apply Theorem 2. Since $\mathrm{Gap}(\bar{M})=O\left(\frac{1}{m^{4}}\right)$ and $\mathrm{Gap}\left(M_{k}\right)=O\left(\frac{1}{m^{3}}\right)$ for all *k*, we have

$$\begin{array}{l} \mathrm{Gap}(M)\geq \frac{1}{2}\mathrm{Gap}(\bar{M})\underset{k\in [m/2]}{\min}\mathrm{Gap}\left(M_{k}\right)\\ \;=\frac{1}{2O\left(m^{4}\right)O\left(m^{3}\right)}\\ \;=\frac{1}{O\left(m^{7}\right)}, \end{array}$$

establishing Theorem 4. □

Finally, an application of Lemma 3 allows us to conclude that the mixing time is also polynomially-bounded.

#### Corollary 1.

M is rapidly mixing.

#### Proof.

In order to apply Lemma 3, we need to obtain a polynomial bound on log(1/*π*(*x*)) for all *x* ∈ Ω. Let *t* ∈ Ω have maximum energy among all elements of Ω. For any *x* ∈ Ω,

$$\begin{array}{l} \log\left(\frac{1}{\pi (x)}\right)=\log\left(\frac{\sum_{y\in \Omega }e^{-\alpha d_{0}(y)-\beta d_{1}(y)}}{e^{-\alpha d_{0}(x)-\beta d_{1}(x)}}\right)\\ \;\leq \log\left(\frac{C_{n}e^{-\alpha d_{0}(t)-\beta d_{1}(t)}}{e^{-\alpha d_{0}(x)-\beta d_{1}(x)}}\right)\\ \;\leq \log\left(\frac{C_{n}e^{-\alpha n-\beta n}}{e^{-\alpha }}\right)\\ \;=\log\left(C_{n}e^{-\alpha (n-1)}e^{-\beta n}\right)\\ \;\leq n\log(2n)+\log\left(\frac{1}{n\;+\;1}\right)-\alpha (n-1)-\beta n. \end{array}$$

This gives us the required polynomial bound, and therefore Lemma 3 implies that M is rapidly mixing. □

## 4. Discussion and Conclusions

The goal of this work was to identify a Markov chain and construct a corresponding algorithm by which to examine the non-uniform distribution and dispersion properties of NNTM RNA secondary structures and branching properties independent of a specific nucleotide sequence. This study successfully identifies the existence of a Markov chain, with a provably polynomial mixing time, which generates a Gibbs distribution on plane trees. This stationary probability distribution models branching characteristics of RNA secondary structure under the NNTM. While the exploration of sampled structures obtained from this algorithm are beyond the scope of the presented results, pseudocode (see Section 3.1) is provided to facilitate future work in this area. Below we discuss the direct applications and implications of this work to RNA modeling, the possibility of implementing a dynamic programming approach, the possibility of an approach using stochastic context-free grammars, other biological applications of this work, contributions of this work towards independent mathematical research interests, and limitations and future directions of the present work.

### 4.1. Applications to RNA Modeling

The most straightforward application of this work is in understanding the background distribution of the branching behavior for secondary structures predicted under the NNTM. While the NNTM is widely used to predict secondary structures from sequence data, little is known about the general branching characteristics of the predicted structures, independent of a specific input sequence. Quantities such as the number of hairpins, the maximum branching in a multiloop, the average branching in a multiloop, and the maximum ladder distance of the structure [7,35] help to characterize the branching behavior and could be computed from samples obtained from this algorithm. These quantities also have been studied in native structures and/or could be easily obtained from databases such as RNA STRAND [36]. The parameter values of *α*, *β*, and *γ* corresponding to various revisions of the NNTM are given in Table 1 in Section 2.1. The Markov chain and corresponding algorithms presented will enable biologists to calculate the dispersion of key branching properties for a specific energy function. As described with the detailed hairpin dispersion example in the Introduction (Section 1), knowing whether branching properties fall within acceptable dispersion limits is crucial for deducing potential functional insight or hypothesizing other scientific ramifications.

Another key application to RNA modeling of the presented algorithms is the ability to explore the parameter space of possible values for *α* and *β*. While the various revisions of the NNTM correspond to specific values for these parameters, in principle any real-valued parameters could be used. Finding values for these parameters that approximate reality remains an open question. Yet, determination of how differences in parameter values change the distribution of NNTM branching properties, such as maximum ladder distance, is crucial. Moreover, parameter space exploration is necessary to identify and further explore the phase transitions that exist. The presented Markov chain and corresponding algorithms expedite such future computational experimentation. Therefore, collectively, the presented algorithm enables exploration that will greatly improve understanding of NNTM-based RNA secondary structures and branching properties, as well as identify potential limitations or specific branching structures where the NNTM models do not sufficiently emulate reality. For example, NNTM-based free energy minimization algorithms achieved an accuracy of at least 60% in only 9% of 16S secondary structures analyzed by Doshi et. al. [15].

The algorithm presented here can only sample under an energy function of the form *αd*_0_ + *βd*_1_, and this does not capture the entirety of the model presented in [16], which considers energy functions of the form *αd*_0_ + *βd*_1_ + *γr*. However, the missing term, *γr*, represents the energy contribution of the exterior loop, and the exterior loop contributes less of the total free energy as sequence length increases. Therefore, when interested in sequences of at least moderate length, this algorithm may be able to provide insight, as long as information about the exterior loop is not the specific object of study. Note that other authors have made similar simplifications with respect to the exterior loop, e.g., [17].

### 4.2. Possibility of a Dynamic Programming Approach

This sampling problem to calculate the dispersion of NNTM RNA secondary structure and properties utilized Markov chain techniques. However, is it possible to utilize a dynamic programming algorithm? It is straightforward to sample Dyck paths under a uniform probability distribution using dynamic programming techniques. However, it is not clear whether a similar technique could be used for the Gibbs distribution we define here, due to the complexity of the energy function. In particular, large numeric computations may be required to handle the variable *k*, the number of *U* steps in a path. While Alonso presents a way to sample from the unweighted distribution $\mathrm{Pr}(k=l)\propto \left(\begin{array}{l} m\\ 2l \end{array}\right)C_{l}$ time without large computations [37], it is unclear if a similar method may be used for the present application.

### 4.3. Possibility of an SCFG Approach

Stochastic context-free grammars (SCFGs) have been widely used in the field of RNA secondary structure prediction, e.g., [38–41]. Most commonly, the probabilities for production rules in an SCFG are determined by training on a set of known secondary structures, often including covariance information from homologous structures. These approaches are not immediately applicable to the problem we study here, as they do not give any insight into the NNTM multiloop energy parameters.

However, some authors have constructed SCFGs based on the NNTM. In particular, Nebel and Scheid [38] construct an SCFG with 29 distinct production rules to mirror the NNTM features. They also present a sampling algorithm allowing for sampling structures of a fixed size using the grammar. However, they do not actually compute probabilities for the production rules that would allow one to sample from a Gibbs distribution (with NNTM energy) and instead rely on training on a set of known structures. Indeed, it is not clear from the paper whether such a set of probabilities must exist.

Even in the case of the simplified model we present in this manuscript, it is not clear how to assign probabilities to production rules in an SCFG so that the probability of obtaining a given structure matches the Gibbs probability under the NNTM. See Supplement 1: SCFG for more details.

Even if a suitable SCFG could be formulated, the SCFG approach is not necessarily superior. The sampling algorithm presented by Nebel and Scheid has time complexity *O*(*n*^3^) and space complexity *O*(*n*^2^). While the algorithm we present does have large time complexity, it only requires linear space, which may be an advantage for some applications.

Even though we cannot easily formulate a SCFG, it is reasonable to consider whether a context-free grammar (such as that presented in Supplement 1) could nonetheless be used as the basis for a dynamic programming algorithm. In fact, this is possible. The key idea is to create a table for each non-terminal symbol *X* and then populate entry *k* of the table with

$$\sum e^{E(t)},$$

where the sum is taken over all trees $t\in T_{k}$ which can be derived from symbol *X*.

Once the tables been populated with these (non-normalized) probabilities, a stochastic backtracking procedure can be used to obtain samples.

However, as in Section 4.2, an assumption that each arithmetic operation can be performed in unit time is not appropriate here. Because the elements of our dynamic programming tables are in fact parts of the partition function, we can conclude that the numbers involved could have up to *O*(*n*) digits. Each arithmetic operation, therefore, becomes much more expensive. While a polynomial-time dynamic programming algorithm based on a context-free grammar is possible, an efficient dynamic programming algorithm would require substantially more work.

### 4.4. Extended Applications

The Markov chain mixing analysis techniques explored in this manuscript have the potential for useful application in a variety of fields. Markov chain Monte Carlo algorithms are widely used in several fields including, machine learning [42], econometrics [43], and Bayesian Statistics [44]. In virtually all applications, an understanding of mixing time increases confidence in the results. In some situations, an understanding of mixing time may also allow for more efficient algorithm selection and implementation.

While many Markov chains with nonuniform stationary distributions have been used for biological applications (e.g., [45–48]), theoretical guarantees on the mixing time are generally not known. Instead, researchers must rely on convergence heuristics, and in fact, many introductions to Markov chain Monte Carlo written for biologists explain such heuristic techniques [49–52]. Of course, heuristics can be misleading, and rigorous mixing time guarantees would be significantly preferable. The same techniques used in this work might be used to generate algorithms with rigorous mixing time bounds for other biological problems concerning a nonuniform distribution.

The mathematical techniques used in this manuscript have been widely used in mathematics, physics, and computer science, demonstrating their broader applicability. For numerous examples, we direct the reader to the books of Levin, Peres, and Wilmer [53]; Montenegro and Tetali [54]; and Jerrum [55].

As an example where similar techniques have found utility in biological applications, it is interesting to briefly consider the study of cladograms, which arise from phylogenetic trees. Mathematically, a cladogram is a binary tree with *n* labeled leaves and *n* − 2 unlabeled internal nodes. While an explicit formula is known for the exact number of cladograms of a given size, mixing time under certain dynamics has also been studied. For example, Aldous [56] studied a Markov chain where a leaf is removed at random and then attached to a random edge in the tree, obtaining a proof that the mixing time is bounded below by *O*(*n*^2^) and bounded above by *O*(*n*^3^). Further work by Schweinsberg [57] later proved an upper bound of *O*(*n*^2^), closing the gap between the upper and lower bounds.

### 4.5. Independent Mathematical Research Interests

The plane trees examined as a model for RNA secondary structure are of independent mathematical interest. As Catalan objects, they have been studied combinatorially (see, for example, [25,58]), and Markov chains on Catalan objects have received significant attention over the years [33,34,59–61], but with very few results providing tight estimates on the corresponding mixing times; most commonly these are discussed in the language of Dyck paths. Cohen’s thesis [33] gives an overview of the known mixing time results for chains on Catalan objects. All of the chains surveyed there have a uniform distribution over the Catalan-sized state space as their stationary distribution. Among these, essentially the only known chain with tight bounds (upper and lower bounds differing by a small multiplicative constant) is due to Wilson [34] and gives the relaxation time of *O*(*n*^3^) for the walk consisting of adjacent transpositions on Dyck paths. In comparison, in [59] the chain using all (allowed) transpositions has been shown to have relaxation time of *O*(*n*^2^), and further conjectured to have *O*(*n*) as the relaxation time, in analogy with the random transposition shuffle of *n* cards.

Judging from the lack of progress on several of these chains, it is evident that determining mixing or relaxation time for these chains is typically a challenging problem, even in the case where the stationary distribution is uniform.

In the current work, the RNA secondary structure modeling naturally leads to a state-space on Catalan objects with a nonuniform distribution, making the corresponding mixing time analysis even more challenging. Another example where mixing times are estimated for Markov chains on Catalan objects with nonuniform stationary distribution is the work of Martin and Randall [30], which examines a Gibbs distribution on Dyck paths weighted by the number of returns to the *x*-axis.

### 4.6. Limitations and Future Directions

While the mixing time proved here is polynomial, it is almost certainly too large to allow for any practical computational sampling experiments. However, we conjecture the actual mixing time to be much smaller, and future work may provide a better bound. Even without additional theoretical results, interesting work is possible using the algorithm we present and heuristic methods for evaluating Markov chain mixing. See ([62], Ch. 8) for a discussion of heuristic methods for monitoring Markov chain convergence.

The results of this study provide an important mathematical foundation for examining the dispersion of RNA secondary structures and branching properties using a Markov chain. However, more work is necessary to optimize the developed computational application for incorporation into the software utilized by biologists that study RNA. Example questions that strongly compel further investigation include:

1. Can the mixing time bound in our main result be improved?
2. Is there a rapidly mixing chain, with the same stationary distribution studied here, whose transitions correspond naturally to moves on the set plane trees? Mixing time bounds on the chain of matching exchange moves, as defined in [63], would be especially interesting, as such a chain may relate to RNA folding kinetics.
3. Is there a rapidly mixing chain converging to the Gibbs distribution using the full energy function for the utilized NNTM model [16]? The chain presented here uses only the parameters *α* and *β*, setting *γ* = 0.
4. Is there a stochastic context-free grammar which generates secondary structures (in our simplified model or using the full NNTM) according to a Gibbs distribution with NNTM energy?

## Supplementary Material

Supplement

## Figures and Tables

**Table 1.** Nearest Neighbor Thermodynamic Model (NNTM) parameters and resulting energy functions. Energy functions are of the form *αd_0_* + *βd*_1_ + *γr*.

Y	Z	Turner	a	b	c	h	f	i	g	*α*	*β*	*γ*
C	G	89	4.6	0.4	0.1	−10.9	3.8	3.0	−1.6	−0.9	−1.8	−1.7
G	C	89	4.6	0.4	0.1	−16.5	3.5	3.0	−1.9	−0.9	−1.2	−1.7
C	G	99	3.4	0	0.4	−12.9	4.5	2.3	−1.6	2.3	1.3	−0.4
G	C	99	3.4	0	0.4	−16.9	4.1	2.3	−1.9	2.2	1.9	−0.4
C	G	04	9.3	0	−0.9	−12.9	4.5	2.3	−1.1	−2.8	−3.0	0.9
G	C	04	9.3	0	−0.9	−16.9	4.1	2.3	−1.5	−2.8	−2.2	0.9

## Abbreviations

The following abbreviations are used in this manuscript:

RNA: ribonucleic acid
NNTM: Nearest Neighbor Thermodynamic Model
SCFG: stochastic context free grammar
