# Supplementary material for: Markov Chain-Based Sampling for Exploring RNA Secondary Structure under the Nearest Neighbor Thermodynamic Model and Extended Applications
Source: Math Comput Appl. Author manuscript; Available in PMC 2022 Aug 2. (PMC9344895; doi:10.3390/mca25040067)
Supplement: Supplement [file NIHMS1820213-supplement-Supplement.pdf]

Anna Kirkpatrick, Kalen Patton, Prasad Tetali, and Cassie Mitchell

Supplement 1: SCFG

1 A stochastic context free grammar

This document attempts to illustrate it is not apparent how to formulate stochastic context free grammar based on the work of Rivas and Eddy (1999) and Nebel and Scheid (2011) that generates a given NNTM Gibbs distribution. To do so, we will attempt to formulate such a grammar, making the most natural or logical choices at each step. We do not claim that finding such a grammar is impossible; we only claim that is not apparent from the existing literature.

We first give a description of a stochastic context free grammar similar to that described by Nebel and Scheid (2011) but restricted to the plane tree model of RNA secondary structure. Notably, each of the production rules corresponds to a specific change in free energy under the NNTM.

Plane tree are represented as strings of parathensies, using the typical Catalan bijection.

The notation for the free energy is consistent with section 2.1 of the submitted manuscript.

The alphabet of terminal symbols is $\{(,)\}$, and the non-terminals are s, t, u, with s being the initial state. We additionally use ϵ to denote the empty string.

For the time being, we leave the probabilities undetermined.

**probability**	**production rule**	**description**	**free energy**
s_1	$s \to (t)s$	branch on exterior loop	g
s_2	$s \to \epsilon$	end of exterior loop	0
t_1	$t \to (t)u$	first branch on a multiloop	$a + 8b + 2c$
t_2	$t \to (t)$	internal node	i
t_3	$t \to \epsilon$	hairpin	f
u_1	$u \to (t)u$	additional branches on a multiloop	$4b + c$
u_2	$u \to (t)$	last branch on a multiloop	$4b + c$

2 Determination of production rule probabilities

Given specific values for the free energy parameters a, b, c, f, g, i, we need to pick specific values for the production rule probabilities $s_1, s_2, t_1, t_2, t_3, u_1, u_2$ so that the probability of generating a given string with the grammar, given the length of the string, is the Gibbs probability.

That is, given a plane tree with n edges, root degree r, and down degree sequence (excluding the root) $d_0, d_1, \ldots, d_{n-1}$, the free energy should be given by

$$gr + id_1 + fd_0 + \sum_{i=2}^{n-1} \left(d_i(a + 8b + 2c) + d_i(i-1)(4b + c) \right),$$

and hence the probability (given the number of edges n) should be

$$\frac{e^{-\left(gr+id_1+fd_0+\sum_{i=2}^{n-1}(d_i(a+8b+2c)+d_i(i-1)(4b+c))\right)}}{Z_n}, \tag{1}$$

where Z_n is the appropriate normalizing constant:

$$Z_n = \sum_{t\in\mathcal{T}_n} e^{-\left(gr(t)+id_1(t)+fd_0(t)+\sum_{i=2}^{n-1}(d_i(t)(a+8b+2c)+d_i(i-1)(4b+c))\right)}.$$

Note that a plane tree with n edges, degree sequence $d_0, d_1, \ldots, d_{n-1}$, and root degree r has probability in the grammar

$$s_1^r s_2 t_2^{d_1} t_3^{d_0} \prod_{i=2}^{n-1} t_1^{d_i} u_2^{d_i} u_1^{(i-1)d_i}.$$

Conditioning on the requirement that the length of the string be $2n$ (or, equivalently, that the plane tree has n edges) gives

$$\frac{s_1^r s_2 t_2^{d_1} t_3^{d_0} \prod_{i=2}^{n-1} t_1^{d_i} u_2^{d_i} u_1^{(i-1)d_i}}{Y_n}, \tag{2}$$

where Y_n is the probability that the grammar generates a string of length $2n$.

Based on the similarity between Equations 1 and 2, it is natural to try setting

$$\begin{aligned}
s_1 &= e^{-g}/Z_s \\
s_2 &= 1/Z_s \\
t_1 &= e^{-(a+8b+2c)}/Z_t \\
t_2 &= e^{-i}/Z_t \\
t_3 &= e^{-f}/Z_t \\
u_1 &= e^{-(4b+c)}/Z_u \\
u_2 &= e^{-(4b+c)}/Z_u,
\end{aligned}$$

where

$$\begin{aligned}
Z_s &= e^{-g} + 1 \\
Z_t &= e^{-(a+8b+2c)} + e^{-i} + e^{-f} \\
Z_u &= 2e^{-(4b+c)}
\end{aligned}$$

are normalizing constants which ensure $s_1 + s_2 = 1$ as well as $t_1 + t_2 + t_3 = 1$ and $u_1 + u_2 = 1$.

We note that, if not for these normalizing constants Z_s, Z_t, Z_u, we would be able to obtain equality between the probability given by the grammar and that given by the Gibbs

distribution. However, the normalizing constants are necessary to satisfy the definition of a stochastic context free grammar.

Proceding with the definitions of $s_1, s_2, t_1, t_2, t_3, u_1, u_2$ given above, we see that the probability that a given tree is generated by the grammar is

$$\frac{s_1^r s_2 t_2^{d_1} t_3^{d_0} \prod_{i=2}^{n-1} t_1^{d_i} u_2^{d_i} u_1^{(i-2)d_i}}{Y_n}$$
$$= \frac{e^{-rg} e^{-d_1 i} e^{-d_0 f}}{Y_n Z_s^{r+1} Z_t^{d_1+d_0}} \prod_{i=2}^{n-1} \left(\frac{e^{d_i(a+8b+2c)} e^{-d_i(4b+c)} e^{-(i-2)d_i(4b+c)}}{Z_t^{d_i} Z_u^{(i-1)d_i}} \right)$$
$$= \frac{e^{-\left(rg+d_1 i+d_0 f+\sum_{i=2}^{n-1}(d_i(a+8b+2c)+d_i(i-1)(4b+c))\right)}}{Y_n Z_s^{r+1} Z_t^n Z_u^{d_0-r}}$$

Note that the numerator now matches exactly with the numerator in the Equation 1. In order to obtain equality for the whole expression, we would need

$$Y_n Z_s^{r+1} Z_t^n Z_u^{d_0-r} = Z_n$$

for all trees with n edges.

The left hand side expands to

$$Y_n \left(e^{-g}+1\right)^{r+1} \left(e^{-(a+8b+2c)} + e^{-i} + e^{-f}\right)^n \left(2e^{-(4b+c)}\right)^{d_0-r}.$$

Even without an explicit expression for Y_n, we know that Y_n is constant for fixed n. However, the non-constant portion

$$\left(e^{-g}+1\right)^{r+1} (2e)^{-(4b+c)(d_{0-r})}$$

clearly varies among trees with n edges. Hence, the denominator we obtain when computing the probability of obtaining a string using the stochastic context free grammar clearly cannot be equal to the constant Z_n.

Therefore, we have shown that one clear approach to formulating a stochastic context free grammar based on the work of Rivas and Eddy (1999) and Nebel and Scheid (2011) fails. We do not claim that no such grammar exists. We only claim that the approach which seems most obvious does not work.
